# Influence of Psychological Perspectives and Demographics on Drivers’ Valuation of Road Accidents: A Combination of Confirmatory Factor Analysis and Preference Heterogeneity Model

**DOI:** 10.3390/bs12090336

**Published:** 2022-09-15

**Authors:** Panuwat Wisutwattanasak, Sajjakaj Jomnonkwao, Chamroeun Se, Vatanavongs Ratanavaraha

**Affiliations:** School of Transportation Engineering, Institute of Engineering, Suranaree University of Technology, Nakhon Ratchasima 30000, Thailand

**Keywords:** willingness-to-pay, psychological perspectives, demographics, confirmatory factor analysis, unobserved heterogeneity

## Abstract

Property damage and loss from road traffic accidents are a major concern in developing countries; thus, studies on accident damage in such countries may include more latent factors. This study aims to examine the effect of psychological perspectives and sociodemographic status on drivers’ willingness-to-pay (WTP) for road accident risk reduction, using confirmatory factor analysis (CFA) and the random parameters multinomial logit model with heterogeneity in means and variances (RPMNLHMV). The CFA results from interviews with 1650 car drivers in Thailand demonstrate that concepts of the theory of planned behavior and health access process approach are key factors for describing drivers’ behavioral intention and WTP. The RPMNLHMV results indicate that drivers’ demographics affected drivers’ WTP to reduce road accidents, and psychological perspectives were also found to have an influence on WTP. The results also reveal unobserved characteristics that could affect drivers’ WTP. The study concludes that ignoring unobserved heterogeneity in studies on WTP to reduce road accidents can lead to biased results and neglect important influential factors. The methodological approaches applied herein offer another layer of insight into unobserved characteristics in road accident valuation. These findings could be used to provide relevant authorities practical insights for policy development on road accident mitigation and road safety education programs in accordance with drivers’ characteristics.

## 1. Introduction

Improved road safety can positively affect individuals’ quality of life and national progress; therefore, many studies attempt to determine the factors that can improve road safety around the world, finding them to be especially effective in developed countries. The issue of traffic accidents has a considerable impact in developing countries [1], reflected by the number of fatalities [2] and substantial loss from road accidents at national levels. Thailand is an appropriate site for investigating these factors and drivers’ attitudes regarding road accident valuation, as the nation is heavily affected by traffic accidents (32.7 fatalities per 100,000 population) [2,3]. Thailand is representative of middle-income developing countries [4] and a center of tourism in the ASEAN economy [5]. The number of traffic-related fatalities indicates that effective road safety improvement in Thailand remains comparably low to that of developed countries; thus, investigating drivers’ perspectives regarding accident damage in Thailand should include more latent factors. In addition to demographic and environmental aspects, studies of human attitudes, risky behaviors, and psychological perspectives related to road accidents have gained increasing attention [6,7,8]. Evidence suggests that the components of accidents and related factors may be influenced by drivers’ thoughts, attitudes [8], and behaviors [9].

In the study of accident reduction valuation, one popular concept is to compare severity in the form of financial or economic losses. This concept supposes that if an accident is very serious, it will result in a high loss as well. Willingness-to-pay (WTP) refers to the maximum expenditure that individuals would consider paying to obtain a product or agree to pay not to lose a product. This concept has been validated as appropriate for research regarding the monetary aspects of road accidents, and therefore is a monetary concept that is widely used in road risk valuation [10]. Where risk is seen as a product, people are willing to pay more if they assess the risk as very dangerous. Many previous studies have used WTP for evaluating road accident risk. Nevertheless, the majority of these studies explored the potential factors that influence individual valuation of road accidents, most of which include socio-demographics [11,12,13], accident experience [14,15], or driving behavior [16,17]. However, from policymakers’ perspective, examining only demographic or environmental factors may be a weak and insufficient approach for developing strategic policies to improve road safety. Consequently, understanding drivers’ perspectives and risk valuations will provide policymakers with more comprehensive insights for the strategic development of road safety improvements. Further, the study of road safety is related to psychological or health behaviors. Several studies have examined drivers’ health and risk behaviors by applying related theories; for example, the health belief model [18,19] and locus of control [20]. However, these concepts are rarely used in investigating drivers’ valuation of road accidents. The value of risk reduction appears to be associated with individuals’ mindset or behavioral intention, as the WTP to reduce accident risk is related to behavioral intention (whether respondents intend to increase safety can be demonstrated by higher WTP [21]. This study determined that applying two psychological concepts, the theory of planned behavior (TPB) and health access process approach (HAPA), can describe behavioral intention to pay and be adopted for investigating drivers’ WTP.

Another aspect to be carefully considered is the analysis method. Most of the WTP studies used traditional standard regression, probit, or logit models, etc. These approaches can only indicate the influencing factors in one layer (i.e., fixed effect of parameter estimates). To explore deeper insights into the effects of factors (layer 2), the concept of unobserved heterogeneity can be applied (introduced by Mannering, et al. [22] in road safety research). The unobserved heterogeneities are the factors that do not directly relate to the dependent variable but act as hidden variables that differently influence the outcome probabilities in the model. Therefore, accounting for unobserved heterogeneity in the modeling process can produce more revealing results in WTP for road accident reduction studies.

In response to the gaps in previous research, the goals of this study were twofold: (1) to understand Thai drivers’ perspectives (using TPB and HAPA), attitudes, sociodemographic status, and experiences that may affect WTP for risk reduction; and (2) to apply a new advanced econometric and statistical approach (i.e., heterogeneity modeling) to uncover insight into the effect of relevant factors on WTP. To achieve the study purposes, we initially applied confirmatory factor analysis (CFA) to confirm the correlations between the relevant indicators of TPB and HAPA and WTP, confirming that these concepts influence drivers’ intention to pay. Subsequently, the CFA results were combined with drivers’ demographics to conduct an in-depth analysis on the factors influencing drivers’ WTP using the random parameter multinomial logit model with heterogeneity in means and variance (RPMNLHMV) to capture variations and unobserved characteristics across drivers, which has not been used in previous studies on WTP for road accidents. The findings provide relevant authorities and policymakers with more comprehensive insights into relevant factors and alternatives for improving road safety in developing countries.

The remainder of this paper is structured into four sections. Section 2 details the related theories and presents a literature review. Section 3 outlines the material and methods used, and Section 4 presents the results analyses and discussion. Finally, Section 5 summarizes the research conclusions and potential directions for future study.

## 2. Literature Review

### 2.1. Psychological Theories

Drivers’ WTP to reduce the risk of road accidents can be represented by behavioral intention. Considerable research examines behavioral intention or health changes as a form of psychological theory to determine drivers’ perspectives on road safety. Consequently, we apply TPB and HAPA theories to capture drivers’ perspectives and behavioral intention as key factors for analyzing their influence on WTP, which are detailed below.

#### 2.1.1. Theory of Planned Behavior

**Figure 1:**
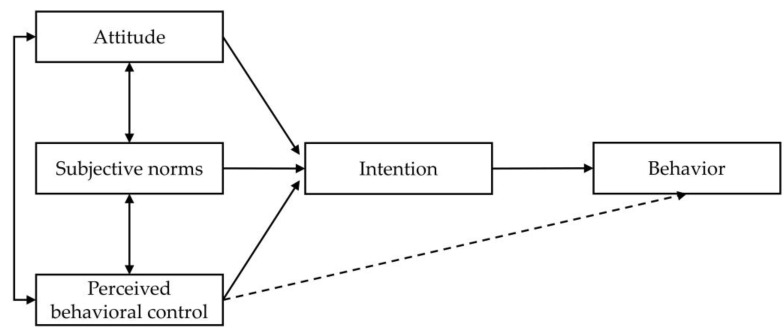
The theory of planned behavior. [23].

The TPB examines individual attitudes and their influence on behavior change and was developed from the concept of the theory of reasoned action [23]. The theory infers that human behavior is influenced by behavioral intention predicted by the three factors of attitude, subjective norms, and perceived behavioral control (see Figure 1). TPB is widely used in behavior studies as it has been validated as a way to explain individuals’ behavioral intention. This study applies this concept to describe drivers’ perspectives regarding these factors’ influence on WTP for road accidents.

#### 2.1.2. Health Access Process Approach

**Figure 2:**
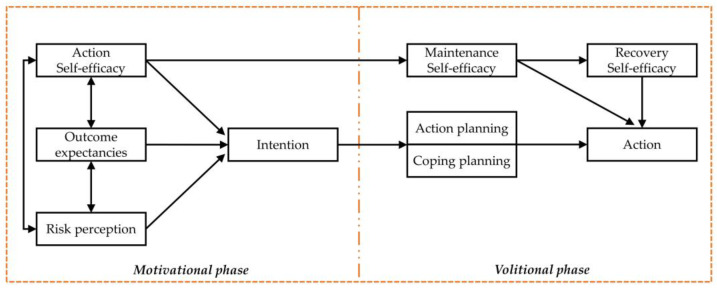
The health action process approach theory. [28].

The HAPA applies theory related to health behavior change [24], focusing on the replacement of usual behaviors to meet health needs. This theory was developed to describe what motivates people to change behavior and explain this process [25,26]. The framework of the HAPA model is divided into two main phases as shown in Figure 2. (1) The motivational phase is a significant aspect because every behavior change begins with intention and motivation, and is comprised of risk perception, outcome expectancies, and self-efficacy (referencing Bandura [27]) (2). The volitional phase includes planning, maintenance self-efficacy, and recovery self-efficacy, which leads to effective action. HAPA has been applied in multiple fields of study but has not been used to investigate drivers’ valuation of road accidents. We apply the motivational phase (behavioral intention) in our study of road accident valuation in conjunction with the direction of the TPB.

### 2.2. Previous Studies on Road Accident Monetary Valuation

Several previous studies regarding drivers’ WTP for road safety improvement apply logit or discrete choice models [14,29,30]. The majority of these studies apply stated choice (SC) survey instruments. This approach asks respondents to consider and compare the utility of specified attributes and identify the alternatives that they consider to be the most cost effective. SC does have some limitations. Since related questionnaires feature closed-ended questions, respondents are unable to indicate WTP in exact values. To address this weakness, we apply the contingent valuation method (CVM), which includes open-ended questions, allowing respondents to identify their exact WTP.

Investigating the potential factors affecting WTP with a discrete choice model is a viable approach, as it allows exploration of the differences among a group of drivers with varying WTP. Subsequently, we construct an advanced discrete choice model with a CVM-based WTP for car drivers, classifying the WTP into three categorical variables: (1) Zero-WTP, which is a group of drivers who are unwilling to pay (specifying the value of WTP = 0); (2) below average WTP; and (3) above average WTP.

The factors associated with previous studies using WTP (20 studies in 14 countries) are presented in Table 1. According to the table, the majority of research only focuses on the respondents’ sociodemographic data, while studies that have introduced the application of psychological theories combined with WTP analysis are rare. This study recognizes the critical role of these concepts on drivers’ decision-making, integrating the TPB and HAPA into the analysis of the factors influencing WTP based on the assumption that behavioral intention regarding risk reduction is influenced by health perspectives and intended behavior. The TPB and HAPA can reveal drivers’ perspectives and attitudes regarding road safety costs. Analyzing the factors affecting WTP to reduce road accidents is complex; therefore, we begin by confirming the components of the related theories using CFA, applying a mixed logit with unobserved heterogeneity to capture the influence of such factors on WTP, including fixed and random parameters, and unobserved characteristics.

## 3. Materials and Methods

### 3.1. Questionnaire Structure

The questionnaire was structured into three main sections. Section 1: driver WTP for road accident risk reduction (using CVM), presenting an open-ended question to obtain numerical values, in which respondents were asked “What is the maximum payment you are willing to pay per 50 km trip to use an improved highway which reduces your chance of fatality or injury from road accident by 50%?” Section 2 collects drivers’ socio-demographics, including gender, age, marital status, income, and education. Accident and driving experience and the purpose of the trip were also gathered, as such information could motivate drivers’ differing perspectives and attitudes. Section 3 introduces questions related to the TPB and HAPA psychological theories to elicit drivers’ opinions and perspectives. The answers in this section are presented on a five-point Likert scale format [45], in which 5 indicates strongly agree and 1 indicates strongly disagree, and the questionnaire was validated using the Item–Objective Congruence test (IOC) (The IOC is a process used to evaluate the content validity in the item development phase, for assessing unidimensional items or items that measure specified composites) [46] with three road safety experts).

### 3.2. Data Collection and Respondent Characteristics

Face-to-face interviews with respondents 18 years or older possessing driver’s licenses in Thailand were conducted to obtain data. Accurate scientific investigation requires that respondents are representative of the population. To ensure representativeness, we included drivers from the four main regions of Thailand (eight provinces with the highest percentage of road accident deaths in each region) via distribution of age, gender, education, income, driving experience, and other considerations, for a total of 1650 respondents. Effectiveness of face-to-face interviews: After the initial screening, all 1650 questionnaires were valid, so none were removed. Our survey was approved by the ethics committee of the Suranaree University of Technology (13 November 2020; COA. 76/2563) and the survey was conducted from 20 November to 13 December 2020. The driver’s characteristics are presented below.

Respondents included 1020 males and 630 females (61.8% and 38.2%, respectively), with an age range of 18–78 (range = 60, mean = 36.33, and standard deviation = 10.67), comprising 752 single drivers (45.6%) and 651 married drivers (39.5%); 48.6% had a bachelor’s degree, 4.3% had a master’s degree, and 0.7% had doctorates; 1011 respondents (61.3%) indicated monthly earnings of 15,000–29,999 baht, 408 respondents (24.7%) reported incomes of 30,000 baht or above, and 14% for the rest. For household income, 319 respondents stated their household income was less than, and 1331 respondents stated their salary was above, 30,000 baht. Of the total 1650 respondents, 245 (14.8%) indicated that they had been in accidents in the past. For the driver’s profession, 79 respondents (4.8%) were students; 175 respondents (10.6%) were government employees; 627 respondents (38.0%) were private companies; 313 respondents (19.0%) owned businesses; and 274 respondents (16.6%) were general laborers. Almost 6 percent, or 293 of the respondents, stated that they usually use their phones while driving. Most of the drivers, or 1615 respondents, had a 5-year license.

### 3.3. Modeling Approaches

#### 3.3.1. Exploratory Factor Analysis and Confirmatory Factor Analysis

We first applied exploratory factor analysis (EFA) to classify the observed indicators of relevant factors. EFA is a technique of factor analysis intended to identify underlying relationships between indicators [47]. Next, we used CFA, which was initially developed by Jöreskog [48], to confirm the correlations among the components obtained from the EFA. The CFA is used to determine whether measures are consistent with the scholarly understanding of the nature of related factors. The purpose of CFA is to test whether the data fit the research hypotheses [49].

#### 3.3.2. Random Parameter Logit with Heterogeneity (in Means and Variance)

We now construct the model within a discrete choice framework wherein the utility function, $U_{ij}$, determines the probability of WTP level *i* obtained from respondent *j* [50], as presented in Equation (1):

(1) $$U_{ij}=\beta_{i}X_{ij}+\varepsilon ,$$

where $\beta_{i}$ enotes the vector for the parameters of WTP level *I*, $X_{ij}$ represents the explanatory variables that affected WTP, and ε is an error component reflecting the unobserved utility component.

Individual-specific unobserved heterogeneity is allowed, and we assume that $\beta_{i}$ has a continuous density function, $Prop\left(\beta_{i}=\beta \right)=f(\beta |\varphi )$, where φ denotes the vector of parameters characterizing this function. The resulting random parameters logit probabilities are calculated with Equation (2) [22]:

(2) $$P_{j}\left(i\right)=\int \frac{EXP\left(\beta_{i}X_{ij}\right)}{\sum_{\forall I}EXP\left(\beta_{i}X_{ij}\right)}f(\beta |\varphi )d\beta ,$$

where $P_{j}\left(i\right)$ is the probability of WTP level *i* associated with respondent *j*, and the other variables are as previously defined. The model is estimated using maximum likelihood estimation with logit probabilities. Accounting for the possibility of heterogeneity in the means and variances of random parameters, $\beta_{ij}$ represents the parameters that vary across respondents, which are derived by Equation (3) [50,51,52]:

(3) $$\beta_{ij}=\beta_{i}+\Omega_{ij}Z_{ij}+\sigma_{ij}EXP\left(\psi_{ij}W_{ij}\right)\epsilon_{ij},$$

where $\beta_{i}$ denotes the mean parameter estimate across all respondents; $Z_{ij}$ represents a vector of explanatory variables capturing heterogeneity in means that influence WTP level *i*; $\Omega_{ij}$ is an estimable parameter vector of $Z_{ij}$; $W_{ij}$ is a vector of WTP variables capturing the standard deviation of heterogeneity $\sigma_{ij}$, with corresponding vector $\psi_{ij}$, and $\epsilon_{ij}$ is the error term.

### 3.4. Research Framework

**Figure 3:**
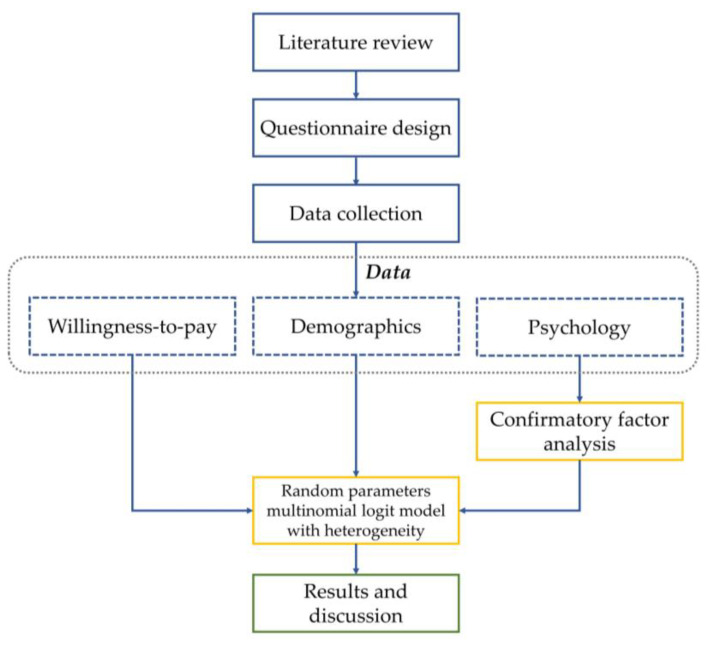
Research procedure.

This study began with a literature review to identify research gaps and weaknesses discovered in previous research and investigate potential statistical methods and theories that could be applied to the WTP study. Next, we developed questionnaires based on review results and collected data from car drivers using face-to-face interviews. Then, two statistical methods (CFA and RPMNLHMV) were applied in sequence to achieve the objectives. First, CFA was used to confirm the measurement indicators of TPB and HAPA. Later, we inputted the WTP, demographics, and CFA results into a RPMNLHMV. Finally, we presented the statistics results and discussion. The research procedure is shown in Figure 3.

## 4. Results and Discussion

### 4.1. Descriptive Statistics and Willingness-to-Pay of Drivers

We categorized the independent variables for statistical analysis into binary and continuous variables, representing drivers’ characteristics and experiences in the mixed logit model, and Likert-scale variables (ranging from 1 to 5) are used in the CFA to examine the correlations between related theories and drivers’ behavioral intention.

The descriptive statistics of 1650 drivers and responses related to the TPB and HAPA are presented in Table 2. Prior to analyzing the CFA, we must examine the descriptive statistics of the scale data to confirm the fitness of data for the analysis. The criteria of skewness were lower than an absolute of 2, kurtosis was an absolute of 7 [49], and the value of Cronbach’s alpha was to be greater than 0.6 [53]. Table 2 demonstrates that the skewness and kurtosis statistics ranged from −0.96 to −0.08, and −1.33 to 1.44, respectively, and Cronbach’s alpha ranged between 0.637 and 0.793; thus, we can conclude that our sample statistics are normally distributed and can be accepted.

Regarding variable coding, to achieve the main purpose of this study (which is to find out what factors are related to the level of WTP and provide policymakers with practical implications for improving road safety accordingly), we decided to classify WTP into three important groups. The first group was Zero-WTP (i.e., drivers indicating WTP = 0). According to the previous studies, most of them found that some respondents indicated their WTP was equal to zero, yet most were omitted from the analysis (e.g., Haddak [39] and Andersson [33]). However, we recognized that respondents who are unwilling to pay are still important from a policymaker’s point of view and may have different attributes. So, it was necessary to separate those who specify WTP = 0 from other groups. The study therefore included them in the analysis to determine what factors affect unwillingness to pay. We also discovered that lower WTP and higher WTP groups had different characteristics and perspectives. Our classification criteria for Low- and High-WTP are based on previous research. That is, a majority of them used the average WTP as a base value to estimate the total cost of road accidents [16,17]. Then, they used linear regression to determine which factors are associated with high WTP (or low WTP). Therefore, it is reasonable to imply that the average WTP can be used as a reference value for WTP classification. As a result, the Low- and High-WTP were classified in this study based on the average (i.e., mean value) of overall WTP. By categorizing WTP into three levels and analyzing it with an advanced logit model, the objective is to allow policy or decision makers to effectively implement the recommended practical implications in accordance with the target groups.

Finally, we defined the dependent variable (WTP) in categories in which 1 denotes drivers indicating WTP = 0 (Zero-WTP), 2 refers to the driver with a WTP greater than 0 but below all drivers’ average (Low-WTP), and 3 represents the remaining driver responses (WTP ≥ overall average; High-WTP). The results reveal that 114 drivers (6.91%) had Zero-WTP for two main reasons: (1) Drivers considered paying for road safety improvement to not be their responsibility, and/or (2) they thought paying for road safety does not elicit demonstrable results. Furthermore, we found that 1114 drivers (67.58%) had Low-WTP, and the remainder (25.52%) exhibited High-WTP. The values of WTP to reduce road accident risk by 50% were approximated at 23 baht per 50 km trip (SD = 16.25 baht).

### 4.2. Exploring the Factor Components and Correlations

#### 4.2.1. The Exploratory Factor Analysis of Observed Factors

We used EFA to define the observed indicators representing the components of each latent factor and computed the primary factors. Table 3 presents the EFA results, identifying 24 items as components of seven latent factors, including risk perception, intention, outcome expectancies, self-efficacy, attitude, subjective norms, and perceived behavioral control. The component loadings ranged between 0.560 and 0.873. The seven factors had construct reliability (CR) ranging from 0.756 to 0.893 and average variance extracted (AVE) was between 0.439 and 0.735. The statistical value of AVE was at least 0.4 and CR not less than 0.7, and therefore they could be accepted in the EFA [58,59,60]. These results confirm that all factors were suitable for CFA.

#### 4.2.2. Theoretical Confirmation

Using the EFA results, this section examines the explanatory power of each item to confirm that indicators can be components of the TPB and HAPA. The CFA results using Mplus 7.2 software by Muthén and Muthén, Los Angeles, CA, USA, illustrates that all indicators were significant as factor components of the TPB and HAPA, with all parameters significant at the 0.01 level. Model fit statistics were $\chi^{2}$ = 469.783; df = 187; $\chi^{2}/df$ = 2.512; CFI = 0.971; TLI = 0.957; SRMR = 0.039; and RMSEA = 0.030. These statistics were in accordance with empirical data compared to acceptance criteria. The model estimation results are presented in Table 4 and discussed below.

According to HAPA, RP1–RP4 are the components of risk perception, and “I know that every time I drive, there is always a chance of road accidents” was the highest indicator. This was followed by the three indicators of outcome expectancies (OE1–OE3), of which “I think that paying for safer roads will give me the benefits I need” represented the most influential factor. And self-efficacy was measured by SE1–SE3, of which “When I drive, it is always easy for me to consider using a safe road” had the highest factor loading.

The latent factors of attitude in the TPB were measured by variables A1–A3, and “Most of my family will perceive me as more safety responsible if I pay more to use a safer road” represented the highest influential factor. This was followed by subjective norms, whose measurement was confirmed using S1–S3, and “Most of my family pays for safe road usage to reduce the chance of road accidents” obtained the highest factor loading. The next was perceived behavioral control, verifying that the three related variables (P1–P3) were valid measures, where “Reducing road accidents can be my control by paying to use a safe road” had the highest factor loading.

Finally, behavioral intention was a component of both the HAPA and TPB models, and our results also validated the four indicators (I1–I4) as measures of driver intention, finding “I will pay more to use a safer road” was the highest influential indicator.

As demonstrated by the results in Table 4, the indicators of each factor are appropriate for significantly measuring TPB and HAPA; therefore, we computed each indicator into the main factors using the beta weight, to reduce the number of factors. Finally, there were the seven remaining constructs of attitude, subjective norms, perceived behavioral control, risk perception, outcome expectancies, self-efficacy, and behavioral intention.

In addition, correlations between constructs are presented in Table 5 to ensure that no pair of factors is overly correlated, referencing Mukaka [61], who asserted that correlations between relevant variables should be less than ±0.750. There is also evidence that the square roots of AVE can present a good explanation of constructs and discriminant validity, as previous studies reported that square roots of AVE of each factor should be greater than the correlation coefficients of their counterparts [58,62], and our results confirm that the statistical values were within the acceptable range.

### 4.3. Factors Influencing Drivers’ Willingness-to-Pay for Road Accident Reduction

#### 4.3.1. Model Estimation Results

From the questionnaire data obtained from drivers, we investigated a total of 32 factors on the demographic status and experience of drivers. But after we initially analyzed the model, four aspects were removed from the model, including profession of driver, phone used, type of license, and household income, because they were not found to have a significant relationship with other variables or the outcome WTP (additionally, inclusion of these factors were not found to improve the model fit statistic). Finally, 28 driver demographic items and 24 psychological items were accepted and presented in Table 2 for the analysis.

Table 6 presents the model statistics and results of the significant factors affecting drivers’ WTP applying the RPMNLHMV with Nlogit6 software. We identified four characteristics that influence drivers indicating the unwillingness to pay for road safety improvement. First, married drivers tend to prefer a WTP of 0, from which we can imply that married Thai drivers have more expenses, resulting in no intention to pay more. Drivers’ income is also found to be an influential factor associated with WTP [13,33]. A salary of at least 15,000 baht falls within Thailand’s middle-income group; thus, drivers with adequate salaries are more likely to pay for safety rather than be reluctant to pay [15] (this finding is also consistent with that of Mon, et al. [42], who discovered that middle-income drivers have a positive effect on WTP). Education was found to influence drivers’ risk valuation. Drivers holding a master’s degree were less likely to express Zero-WTP, presumably because higher education helps individuals to better understand the impacts of road accidents, which is consistent with Yang, et al. [40]. Perceived behavioral control, a factor of TPB, was also found to have a direct effect on WTP. This factor is related to drivers’ self-conception and emotions; therefore, if drivers perceive that the WTP for safety does not exceed capabilities, a resulting WTP will emerge [21,63], which is consistent with the result of Subhan, et al. [64], who stated that individuals who felt they had greater control over their finances were more likely to pay for the improvement in road safety.

Regarding Low-WTP, sole earners had a significant effect on WTP, as sole earners are more likely to express a WTP that is higher than 0, but less than the overall average. This implies that sole-earning drivers are aware of the impact of road accidents that could affect their incomes and productivity [15], recognizing WTP for safety as a superior alternative to the consequences of accidents. Nevertheless, such drivers may face challenges regarding the amount of WTP, as sole earners have considerable responsibilities and expenditures, resulting in a lower WTP.

Regarding High-WTP drivers, the results indicated that drivers who regularly travel at nighttime place higher value on safe behavior. The results reflect the findings of Ackaah, et al. [65] and Champahom, et al. [66], which reported that driving at night is more dangerous and could result in increased accident severity. This offers the logical explanation for the finding of the present study showing that nighttime drivers are more aware of their accident risks, which causes them to prefer a higher value of WTP. The components of HAPA reveal important insights regarding how drivers’ outcome expectancies toward safety improvement have a significant influence on WTP. In this context, we can assert that outcome expectancy refers to drivers’ perceptions of effectiveness [64]. If drivers think that WTP for road safety improvement will effectively reduce road traffic severity, they are more likely to have a High-WTP. Evidence from Gebbers, et al. [57] reveals that outcome expectancies were positively related to the intention to behave. Subjective norms were also found to have a negative influence on High-WTP drivers. This factor is related to personal beliefs regarding social trends. In other words, if a driver’s intimate relations have safe road behavior, drivers will be similarly influenced to engage in the same behavior [21]. As indicated by the marginal effect value (highest compared with all other variables), the finding suggests that drivers with more normative beliefs tend to be in the Low-WTP group. This is logical, and the explanation may be attributed to the fact that most of the sample populations have chosen Low-WTP (as clearly shown by the descriptive statistic in the earlier section). Therefore, people who are more likely to follow social trends are also more likely to fall into the Low-WTP group.

#### 4.3.2. Distribution of Random Parameters

**Figure 4:**
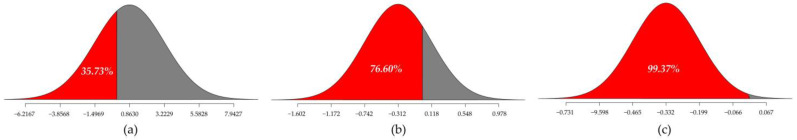
Distribution split of the random parameters. Note: (**a**) gender, (**b**) attitude, and (**c**) annual mileage.

Table 6 also reveals that the significant indicators are random parameters of the models, finding that male drivers and attitude toward risk are random parameters for Low-WTP, and annual mileage is a random parameter of High-WTP drivers. The positive coefficient of the random parameters indicates that the majority of drivers are more likely to fall into the reference group, while the remainder represent other groups (negative coefficients are opposites). Figure 4 presents the proportion percentage between below and above zero of each random parameter (red representing the probability of below zero, and above zero is gray). This study also revealed that most male drivers have at least a Low-WTP, indicating a higher likelihood to pay, as males perceive that their driving behavior makes them a higher risk for accidents than females (consistent with results of Balakrishnan and Karuppanagounder [13] and Yang, et al. [40], who concluded that male drivers have a higher perception of their risk behavior, resulting in a higher WTP). Moreover, results from Andersson [33] showed that female drivers were less likely to pay for safety improvement compared with male drivers. The attitude toward risk in this analysis has an extremely influential role, as drivers with high attitude scores also tend to have High-WTP. In contrast, low attitude scores could affect drivers’ Zero-WTP as well [21]. Evidence from the finding of Subhan, et al. [64] reported that attitude toward traffic safety responsibility was found to be significantly associated with the intention to pay. Similarly, high annual mileage appears to be related to drivers’ preference for lower WTP for road safety. There are two possible reasons for this result. High mileage per year makes drivers more proficient and experienced, and the greater the distance, the greater the cost, resulting in a reduced WTP per trip. This is consistent with the finding of Yang, et al. [40], who also found that highly experienced motorists tend to decrease WTP, as these groups of people are often more experienced and skilled, and they believe that life-threatening events can be avoided by themselves.

#### 4.3.3. Influence of Heterogeneity in the Means and Variances of Random Parameters

While previous WTP studies generally assumed that the mean and variance of the random parameters were fixed (using a standard mixed logit model), this study extensively explored the possibility that explanatory variables have a significant influence on the mean and variance of the random parameters (i.e., shifting the distribution of the random parameters to the left or right and influencing the randomness of the random parameters). Therefore, after the random parameters were found in Table 6, we tested the effect of each non-significant fixed parameter on the mean or variance of the random parameters. As a result, some of the factors (presented below) were found to indirectly affect the outcome probabilities by influencing the mean value and variability of the random parameters (whereas these factors were not significant as fixed-parameters with direct effect). Such circumstances indicate that ignoring this deeper layer of unobserved heterogeneity would indeed result in bias and unreliable results or conclusions.

Table 6 also illustrates the insight characteristics for influencing random parameters; the coefficient of heterogeneity on random parameters indicates that the mean values and variance of each random parameter are influenced by unobserved heterogeneity.

Annual mileage was influenced by young members, compelling trips, and behavioral intention. In general, we determined that higher annual mileage increases drivers’ driving expertise; thus, they are less likely to pay. However, behavioral intention to reduce road accidents and drivers with children will increase the awareness of accidents, resulting in a higher WTP. This is consistent with Svensson and Johansson’s [17] findings that indicated drivers who have children in their family will have more road safety responsibilities, resulting in a higher WTP. Moreover, according to the Subhan, et al. [64] findings, intention plays a significant role in drivers’ road safety awareness. Drivers who have a higher safety intention will have more WTP. In contrast, drivers with compelling trips are more likely to decrease the level of WTP based on the same factor of annual mileage and proficient driving skills. Our results also infer that behavioral intention has a positive influence on drivers’ attitudes toward road accidents and is found to be a factor with variation across groups of drivers. However, we found that behavioral intention in the role of unobserved characteristics can increase the means of drivers’ attitudes and influence safe behavior. As shown in Subhan, et al. [64], intention and attitude toward road accidents were found to represent safety concerns and influenced drivers’ WTP for safety.

Furthermore, the results of heterogeneity in variance revealed that drivers with elders in the household raise the variation in attitude toward road safety improvement, as vulnerable members in the household could increase drivers’ awareness of the severity of road accidents [37].

## 5. Conclusions, Implications, and Research Limitations

This study presented findings from a combination of CFA and RPMNLHMV modeling, revealing insights regarding newly introduced factors (psychological perspectives) on drivers’ WTP to reduce road accidents. Our data were gathered from 1650 car drivers across Thailand using a face-to-face interview questionnaire. This study demonstrated that traditional sociodemographic factors and those of the HAPA and TPB have an influence on driver valuation. Consequently, we reveal significant results by introducing such concerns. Our main conclusions are divided into two main parts (CFA and RPMNLHMV) below.

The initial results of the CFA revealed that all observed indicators are valid measures of TPB and HAPA, and such factors are significantly associated with intention to pay for road accident risk reduction. The results of the correlation demonstrated that self-efficacy, attitude, and perceived behavioral control positively correlate with behavioral intention. In contrast, the factors of drivers’ risk perception, outcome expectancies, and subjective norms had a negative correlation. Further, our study used these factors to conduct more in-depth analysis using a mixed logit model to identify the significance of the factors’ influence on WTP prediction.

Examining demographics using RPMNLHMV demonstrated that married drivers tend toward unwillingness to pay. In contrast, drivers who have middle incomes, a master’s degree, are sole earners, and engage in nighttime travel had a greater than Zero-WTP for road accident reduction. Regarding psychological characteristics, the results indicated that drivers’ outcome expectancies and perceived behavioral control leads to higher WTP. Conversely, subjective norms had a negative effect on WTP. In addition, the three indicators of gender, attitude, and annual mileage were revealed to be random parameters influencing variations in the model. The unobserved characteristics demonstrated that young members and behavioral intention increases the mean of the random parameter, and compelling trips have the opposite effect. Finally, drivers with elder family members in their household increase the variance of attitude toward road accidents.

For policy implementation, our findings revealed the driver characteristics that can affect the WTP as well as provided important insights based on the HAPA and TPB as influences of WTP. These results can benefit relevant authorities when developing road safety guidance, as drivers’ socio-demographics appear to be appropriate considerations in the strategic promotion of road safety education [67,68]. The concept of risk valuation using WTP allows policymakers to identify whether certain characteristics of drivers affect their perception of road accidents. For example, this study found male drivers tend to pay more to reduce risk. This risk may be a result of their driving behavior. Therefore, the relevant authorities should focus on this in training and education on road risk reduction. In addition, nighttime affects the driver’s perception of risk. Therefore, agencies should pay more attention to risk management at night, such as light, traffic, vision etc.

In addition to the demographics and general status, the significance of drivers’ views and mindset are also demonstrated by this study. The TPB and HAPA results indicated that drivers with health awareness who plan specific behaviors are more likely to have higher risk concerns and pay for road safety [56]. These findings can serve as a suitable guideline for policymakers to raise public awareness and attitudes toward road safety. For example, the intention and attitude toward safety had a positive impact on drivers’ risk valuation. Therefore, relevant authorities (such as the Transport Office) should focus on improving drivers’ awareness of the dangers of road accidents by integrating these lessons into driver’s license test programs or safety communication campaigns in order to positively improve their attitudes and behaviors toward road safety. These findings are also confirmed by the literature [69,70]. In addition, outcome expectancies were also found to be important to the driver’s risk valuation. This result formulates the relationship between WTP and road users’ expectations of the effectiveness of government efforts. In simple terms, the government should demonstrate budgeting efficiency by improving road safety. This may increase the probability of drivers’ willingness to pay for safety as they perceive the value of road safety.

In terms of methodological novelty contribution, based on the authors’ review, this study is the first to attempt to extend a random parameters logit model by further allowing the possibility that preference-level variables may have indirect influences on the outcome WTP value probabilities by shifting the means and variances of the random parameters. Evidently, in this study, variables reflecting households with children, driving to work or for work, drivers’ intention, and households with elderly were initially found to be insignificant and would be ignored using the traditional discrete choice models. However, in our heterogeneity modelling approach, these indicators were found to have an effect on the random parameter distributions that have direct influence on the outcome probabilities (for example, the indicator for the elderly had no effect on WTP, but it had a positive influence on the attitude of drivers). This study highlights the importance and necessity of accounting for unobserved heterogeneities in uncovering possible multi-layers of unobserved effects of preference-level (e.g., demographic and psychological) variables on drivers’ WTP for road safety. The proposed approach could offer a more flexible way to fully untangle the effect of significant variables in WTP-related studies.

Among the research limitations, our study only focuses on car drivers, and other types of road users are not included. We also did not include drivers under 18 years old in the study, following Thailand’s licensing law. The inclusion of younger drivers and other road users may have reveal differing perspectives, attitudes toward safety, and knowledge of road safety [71]. Thus, collecting such information will more comprehensively represent the population. Moreover, including environmental factors may be beneficial to road risk valuation research (e.g., road conditions and environmental conditions). These might affect the drivers’ risk perception in accordance with their driving or living area. In addition, future studies could also be conducted in multiple developing nations to provide further relevant insights and data.

## Figures and Tables

**Table 1:** Summary of previous studies on WTP for accident risk reduction and relevant factors.

Author	Factors												Method
	Country	Age	Gender	EXP	Accident	Income	Status	Education	HS	Child	Speed	Psychology	
Persson, et al. [31]	Sweden	✓		✓	✓	✓							Regression
Fauzi, et al. [32]	Malaysia	✓	✓		✓	✓	✓						Regression
Alberini, et al. [11]	Canada	✓	✓					✓					Regression
Andersson [33]	Sweden	✓	✓	✓	✓	✓		✓					Regression
Bhattacharya, et al. [15]	India	✓			✓	✓		✓	✓				Regression
Gibson, et al. [34]	Thailand	✓	✓					✓					Regression
Andersson and Lindberg [35]	Sweden	✓	✓	✓	✓					✓			Regression
Svensson and Johansson [17]	Sweden	✓	✓			✓				✓	✓		Regression
Hoffmann, et al. [12]	Mongolia	✓	✓			✓		✓					Regression
Liu and Zhao [36]	China	✓		✓		✓		✓					Binary logit
Antoniou [14]	Greece	✓	✓		✓		✓						Ordered probit
Robles-Zurita [37]	Spain	✓	✓	✓		✓	✓			✓			Regression
Ainy, et al. [38]	Iran	✓	✓		✓	✓	✓	✓					Regression
Haddak [39]	France	✓	✓		✓	✓	✓	✓					Tobit model
Yang, et al. [40]	China	✓	✓	✓		✓		✓					Mixed logit
Hoffmann, et al. [41]	China	✓				✓		✓					Regression
Mon, et al. [42]	Myanmar	✓	✓		✓	✓	✓	✓	✓		✓		Regression
Flügel, et al. [43]	Norway	✓	✓	✓		✓		✓		✓			Mixed logit
Balakrishnan and Karuppanagounder [13]	India	✓	✓		✓	✓	✓		✓				Binary logit
Widyastuti and Utanaka [44]	Indonesia	✓				✓				✓			Binary logit
This study	Thailand	✓	✓	✓	✓	✓	✓	✓	✓	✓	✓	TPB and HAPA	CFA and RPMNLHMV

Note: EXP = driving experience; Status = marital status; Accident = own accident; HS = household size; TPB = theory of planned behavior; HAPA = health access process approach; CFA = confirmatory factor analysis; RPMNLHMV = random parameters multinomial logit with heterogeneity in means and variances.

**Table 2:** Descriptive statistics of drivers’ socio-demographics and factors associated with the TPB and HAPA (n = 1650).

Code	Descriptions (Binary)		Frequency		Percentage	
	Demographic and factors;					
	Gender (1 if male driver, 0 otherwise)		1020		61.8%	
	Marital status (1 if married, 0 otherwise)		651		39.5%	
	Age 26−35 years (1 if yes, 0 otherwise)		648		39.3%	
	Age 36−45 years (1 if yes, 0 otherwise)		392		23.8%	
	Age above 45 years (1 if yes, 0 otherwise)		341		20.7%	
	Bachelor (1 if Bachelor, 0 otherwise)		802		48.6%	
	Master (1 if Master, 0 otherwise)		71		4.3%	
	Doctoral (1 if Doctoral, 0 otherwise)		13		0.7%	
	INC1 (1 if 15,000 baht ≤ income < 30,000 baht, 0 otherwise)		1011		61.3%	
	INC2 (1 if income ≥ 30,000 baht, 0 otherwise)		408		24.7%	
	Elder (1 if they have elder (Age ≥ 60) in the household excluding respondent, 0 otherwise)		342		20.7%	
	Young (1 if they have children (Age ≤ 18) in the household, 0 otherwise)		388		23.5%	
	Sole earner (1 if yes, 0 otherwise)		885		53.6%	
	Own accident (1 if driver has been involved in a road accident, 0 otherwise)		245		14.8%	
	Family injured (1 if family/close friends have been injured in a road accident, 0 otherwise)		468		28.4%	
	Family died (1 if family/close friends have been died in a road accident, 0 otherwise, 0 otherwise)		164		9.9%	
	Risk perception (1 if driver stated that his/her risk is higher than the average in Thailand, 0 otherwise)		768		46.5%	
	Ticket (orders for traffic violations) (1 if driver has ever been received a ticket, 0 never)		887		53.8%	
	Safety belt usage (1 if often or always, 0 otherwise)		560		33.9%	
	Alcohol (1 if driver has ever been drunk while driving, 0 never)		101		6.1%	
	Driving exceeds speed limit (1 if often or always, 0 otherwise)		1448		87.8%	
	Compelling trip (1 if most of trips are related with the job, 0 otherwise)		955		57.9%	
	Weekday (1 if most of trips are spent on weekday, 0 otherwise)		1100		66.7%	
	Night (1 if most of trips are spent at nighttime, 0 otherwise)		480		29.1%	
**Code**	**Descriptions (Continuous)**	**Adapted from**	**Mean**	**SD**	**SK**	**KU**
	Household size		2.96	1.38	0.31	−0.75
	Number of cars		1.19	0.46	2.00	4.33
	Annual mileage (1000 km)		22.51	11.55	0.60	0.09
	Driving experience (year)		14.11	9.63	0.72	−0.02
ATTI	Attitude (Cronbach’s alpha = 0.782)	Wu and Chen [54]				
A1	Paying for safe roads is useful because it helps me to reduce the chance of road accidents.		4.57	0.57	−0.96	1.14
A2	Paying for safety on road usage makes me feel safer on the road.		4.56	0.57	−0.87	−0.13
A3	Most of my family will perceive me as more safety responsible if I pay more to use a safer road.		4.52	0.60	−0.96	0.33
A4	Most of my friends will perceive me as more safety responsible if I pay more to use a safer road.		4.51	0.62	−0.92	−0.03
SUBJ	Subjective norm (Cronbach’s alpha = 0.793)	Wu and Chen [54], Venkatesh and Davis [55]				
S1	Most of my family pays for safe road usage to reduce the chance of road accidents.		4.15	0.75	−0.28	−1.11
S2	Most of my friends pay for safe road usage to reduce the chance of road accidents.		4.18	0.75	−0.33	−1.12
S3	Most people in my community of friends pay for safe road usage to reduce the chance of road accidents.		4.12	0.78	−0.22	−1.28
PERC	Perceived behavioral control (Cronbach’s alpha = 0.793)	Wu and Chen [54]				
P1	It is my own decision to pay for safe road usage, not depend on others.		4.05	0.77	−0.12	−1.17
P2	Risk of an accident depends on my response. If I pay for a safe road, the chance of road accidents will be decreased.		4.03	0.77	−0.07	−1.28
P3	Reducing road accidents can be in my control by paying to use a safe road.		4.04	0.78	−0.08	−1.33
RISK	Risk perception (Cronbach’s alpha = 0.653)	Ram and Chand [56]				
RP1	I know that every time I drive, there is always a chance of road accidents.		4.16	0.75	−0.29	−1.11
RP2	I perceive that routing factors are one of the causes of road accidents.		4.15	0.78	−0.26	−1.29
RP3	I perceive that road accidents do not only depend on me.		4.14	0.75	−0.25	−1.13
RP4	I perceive the risk of road accidents is inevitable.		4.15	0.75	−0.26	−1.21
OUTC	Outcome expectancies (Cronbach’s alpha = 0.637)	Gebbers, et al. [57]				
OE1	I think that paying for safer roads will give me the benefits I need.		4.11	0.73	−0.17	−1.09
OE2	I know that if I am willing to pay more, I will become safer.		4.08	0.72	−0.13	−1.08
OE3	I continue using safe roads with the rationale that “I will always get what I expect which is reasonable for the money I pay”.		4.29	0.70	−0.46	−0.88
SELF	Self-efficacy (Cronbach’s alpha = 0.708)	Gebbers, et al. [57]				
SE1	When I drive, it is always easy for me to consider using a safe road.		4.50	0.62	−0.85	−0.30
SE2	Even if I drive on an unsafe route only once, I will recognize that I have more chances of a road accident.		4.50	0.62	−0.85	−0.29
SE3	Seeing others pay for safe roads I think I also can do it.		4.44	0.67	−0.78	−0.51
INT	Intention (Cronbach’s alpha = 0.732)	Wu and Chen [54], Venkatesh and Davis [55], Gebbers, et al. [57]				
I1	I will pay more to use a safer road.		4.35	0.68	−0.58	−0.71
I2	I will pay for using the safer road because I believe that it could save my life.		4.30	0.72	−0.57	−0.69
I3	I will recommend my close friends to pay for safe roads to reduce the chance of road accidents.		4.48	0.63	−0.85	0.15
I4	I have planned to pay for using safe roads to reduce road accident risk.		4.51	0.61	−0.90	−0.05

Note: SD = standard deviation; SK = skewness; KU = kurtosis.

**Table 3:** Component loading of related factors.

Code	Component Loadings							CR	AVE
	1	2	3	4	5	6	7		
A1					0.560			0.756	0.439
A2					0.713				
A3					0.706				
A4					0.659				
S1						0.708		0.783	0.546
S2						0.736			
S3						0.771			
P1							0.833	0.893	0.735
P2							0.865		
P3							0.873		
I1		0.735						0.791	0.486
I2		0.681							
I3		0.683							
I4		0.689							
RP1	0.752							0.782	0.473
RP2	0.666								
RP3	0.633								
RP4	0.695								
OE1			0.739					0.792	0.561
OE2			0.810						
OE3			0.693						
SE1				0.731				0.767	0.523
SE2				0.703					
SE3				0.736					

Note: CR = construct reliability; AVE = average variance extracted; Kaiser–Meyer–Olkin = 0.832, Components: 1 = Risk perception; 2 = Behavioral intention; 3 = Outcome expectancies; 4 = Self-efficacy; 5 = Attitude; 6 = Subjective norms; and 7 = Perceived behavioral control.

**Table 4:** Model results of confirmatory factor analysis.

Code	Description	Estimates	S.E.	*t*-Stat
ATTI	Attitude;			
A1	Paying for safe roads is useful because it helps me to reduce the chance of road accidents.	0.346	0.029	11.954
A2	Paying for safety on road usage makes me feel safer on the road.	0.481	0.029	16.504
A3	Most of my family will perceive me as more safety responsible if I pay more to use a safer road.	0.586	0.028	21.034
A4	Most of my friends will perceive me as more safety responsible if I pay more to use a safer road.	0.499	0.028	18.035
SUBJ	Subjective norm;			
S1	Most of my family pays for safe road usage to reduce the chance of road accidents.	0.549	0.024	23.191
S2	Most of my friends pay for safe road usage to reduce the chance of road accidents.	0.468	0.025	18.377
S3	Most people in my community of friends pay for safe road usage to reduce the chance of road accidents.	0.544	0.025	22.123
PERC	Perceived behavioral control;			
P1	It is my own decision to pay for safe road usage, not depend on others.	0.721	0.014	50.602
P2	Risk of an accident depends on my response. If I pay for a safe road, the chance of road accidents will be decreased.	0.798	0.012	63.872
P3	Reducing road accidents can be in my control by paying to use a safe road.	0.804	0.012	64.754
RISK	Risk perception;			
RP1	I know that every time I drive, there is always a chance of road accidents.	0.603	0.023	26.281
RP2	I perceive that routing factors are one of the causes of road accidents.	0.475	0.025	18.855
RP3	I perceive that road accidents do not only depend on me.	0.510	0.023	22.510
RP4	I perceive the risk of road accidents is inevitable.	0.550	0.023	23.665
OUTC	Outcome expectancies;			
OE1	I think that paying for safer roads will give me the benefits I need.	0.695	0.030	23.213
OE2	I know that if I am willing to pay more, I will become safer.	0.586	0.025	23.142
OE3	I continue using safe roads with the rationale that “I will always get what I expect which is reasonable for the money I pay”.	0.688	0.034	20.492
SELF	Self-efficacy;			
SE1	When I drive, it is always easy for me to consider using a safe road.	0.582	0.030	19.508
SE2	Even if I drive on an unsafe route only once, I will recognize that I have more chances in a road accident.	0.568	0.030	19.135
SE3	Seeing others pay for safe roads I think I also can do it.	0.482	0.029	16.860
INT	Intention;			
I1	I will pay more to use a safer road.	0.777	0.020	38.722
I2	I will pay for using the safer road because I believe that it could save my life.	0.626	0.020	30.855
I3	I will recommend my close friends to pay for safe roads to reduce the chance of road accidents.	0.423	0.024	17.286
I4	I have planned to pay for using safe roads to reduce road accident risk.	0.364	0.026	14.148

**Table 5:** Correlations between constructs and discriminant validity.

$\sqrt{\mathrm{AVE}}$	INT	RISK	OUTC	SELF	ATTI	SUBJ	PERC
**INT**	**0.697**						
**RISK**	−0.117 **	**0.688**					
**OUTC**	−0.205 **	0.002	**0.749**				
**SELF**	0.179 **	0.089 **	0.079 **	**0.723**			
**ATTI**	0.245 **	0.161 **	0.134 **	0.255 **	**0.662**		
**SUBJ**	−0.116 **	0.582 **	−0.019	0.108 **	0.116 **	**0.739**	
**PERC**	0.323 **	−0.492 **	0.257 **	0.124 **	0.135 **	−0.511 **	**0.857**

Note: ** indicates that correlation is significant at 0.01 level (2-tailed). Square roots of AVE are presented **in bold** in the diagonal row.

**Table 6:** Model results of random parameter logit model with heterogeneity in means and variance.

Variables	Coefficients	*p*-Value	*t*-Stat	Marginal Effect		
				Zero-WTP	Low-WTP	High-WTP
Constants [ZW]	5.100	*	1.75			
Constants [HW]	6.136	**	2.29			
Non-random parameter;						
Marital status (married) [ZW]	0.574	*	1.70	0.0109	−0.0073	−0.0036
15,000 baht ≤ Income < 30,000 baht [ZW]	−0.953	**	−2.05	−0.0224	0.0153	0.0070
Perceived behavioral control [ZW]	−0.924	***	−2.72	−0.1428	0.0945	0.0483
Master degree [ZW]	−2.278	*	−1.79	−0.0014	0.0009	0.0005
Sole earner [LW]	0.551	*	1.79	−0.0072	0.0238	−0.0166
Night [HW]	0.649	*	1.75	−0.0026	−0.0111	0.0137
Outcome expectancies [HW]	0.797	**	2.33	−0.0452	−0.1928	0.2380
Subjective norm [HW]	−1.900	***	−3.84	0.1080	0.4378	−0.5458
Random parameter; (normal distribution)						
Gender (male) [LW]	0.863		0.38	0.0114	−0.0082	−0.0032
Standard deviation	2.360	**	2.05			
Attitude [LW]	−0.312		−0.55	−0.0200	0.1329	−0.1130
Standard deviation	0.430	*	1.90			
Annual mileage [HW]	−0.332	***	−2.80	0.0230	0.0192	−0.0422
Standard deviation	0.133	***	2.81			
Heterogeneity in means;						
Annual mileage: Young	0.063	**	2.27			
Annual mileage: Compelling trip	−0.047	**	−2.11			
Annual mileage: Intention	0.058	**	2.55			
Attitude: Intention	0.180	*	1.75			
Heterogeneity in the variance;						
Attitude: Elder	0.955	*	1.85			
Model statistics;						
Halton draw	1000					
Number of observations	1650					
Number of estimated parameters (K)	48					
Log-likelihood at zero, LL(0)	−1812.710					
Log-likelihood at convergence, LL(β)	−1205.913					
Adjusted ρ^2^	0.308					
AIC_c_	2510.765					

Note: * *p* < 0.1; ** *p* < 0.05; *** *p* < 0.01. ZW = Zero-WTP; LW = Low-WTP; HW = High-WTP.

## Data Availability

Data are available on request from the authors.
